# NGF-Dependent and BDNF-Dependent DRG Sensory Neurons Deploy Distinct Degenerative Signaling Mechanisms

**DOI:** 10.1523/ENEURO.0277-20.2020

**Published:** 2021-01-21

**Authors:** Andrés de León, Julien Gibon, Philip A. Barker

**Affiliations:** 1 Department of Neurology and Neurosurgery, Montreal Neurological Institute, McGill University, Montreal, Quebec H3A 2B4, Canada; 2 Department of Biology, University of British Columbia Okanagan, Kelowna, British Columbia V1V 1V7, Canada

**Keywords:** axons, BAX, BDNF, degeneration, dorsal root ganglion, NGF

## Abstract

The nerve growth factor (NGF) and brain-derived neurotrophic factor (BDNF) are trophic factors required by distinct population of sensory neurons during development of the nervous system. Neurons that fail to receive appropriate trophic support are lost during this period of naturally occurring cell death. In the last decade, our understanding of the signaling pathways regulating neuronal death following NGF deprivation has advanced substantially. However, the signaling mechanisms promoting BDNF deprivation-induced sensory neuron degeneration are largely unknown. Using a well-established *in vitro* culture model of dorsal root ganglion (DRG), we have examined degeneration mechanisms triggered on BDNF withdrawal in sensory neurons. Our results indicate differences and similarities between the molecular signaling pathways behind NGF and BDNF deprivation-induced death. For instance, we observed that the inhibition of Trk receptors (K252a), PKC (Gö6976), protein translation (cycloheximide; CHX), or caspases (zVAD-fmk) provides protection from NGF deprivation-induced death but not from degeneration evoked by BDNF-withdrawal. Interestingly, degeneration of BDNF-dependent sensory neurons requires BAX and appears to rely on reactive oxygen species (ROS) generation rather than caspases to induce degeneration. These results highlight the complexity and divergence of mechanisms regulating developmental sensory neuron death.

## Significance Statement

The elimination of neuronal cells generated in excess during embryonic stages characterizes the maturation of the nervous system. Here, we address the developmental cell death mechanisms of brain-derived neurotrophic factor (BDNF)-dependent dorsal root ganglion (DRG) neurons *in vitro*, comparing and contrast them with those deployed in NGF-dependent sensory neurons. We observe several important differences between the molecular signaling pathways behind nerve growth factor (NGF) and BDNF deprivation-induced death. Significantly, degeneration of BDNF-dependent sensory neurons requires BAX but not caspase activation, instead reactive oxygen species (ROS) generation appears to play a key role in degeneration. This work highlights the complexity of cell death mechanisms in distinct embryonic sensory neuron populations.

## Introduction

The developing nervous system undergoes a period of neuronal cell death during embryogenesis (Patel et al., 2000; Buss et al., 2006; Schuldiner and Yaron, 2015). In this period, neurons that fail to receive trophic support die by apoptosis (Burek and Oppenheim, 1996), a type of cell death also commonly observed in neurodegenerative diseases (Kirkland and Franklin, 2003; Fischer and Glass, 2007; Saxena and Caroni, 2007; Vickers et al., 2009; Tait and Green, 2010; Kanaan et al., 2013). The mammalian peripheral nervous system (PNS) offers a well-characterized context to study developmental neuronal apoptosis. Diverse sub-types of sympathetic and sensory neurons develop, compete, survive, or die based on their capacity to bind enough trophic support from their target tissue (Barde, 1989; Saxena and Caroni, 2007).

Neurotrophins are crucial regulators of survival during the development of the nervous system. Alterations of their levels induce dramatic changes of innervation in the adult PNS (Levi-Montalcini and Booker, 1960; Ernfors et al., 1994a,b; Tessarollo et al., 1997). In mammals, the neurotrophin family is composed of the nerve growth factor (NGF), brain-derived neurotrophic factor (BDNF), neurotrophin-3 (NT3), and neurotrophin-4/5 (NT4/5). With equal low affinity and no selectivity, each neurotrophins can bind to the pan-neurotrophin receptor p75 (p75NTR), and with high affinity to the tropomyosin-related kinase (Trk) receptor family: with NGF binding to TrkA, BDNF and NT4/5 to TrkB and NT3 to TrkC. Sympathetic and sensory neurons can be classified based on their expression profile of Trk receptors and their requirement for neurotrophins. Most sympathetic and sensory neurons depend on the NGF-TrkA signaling pathway during development (Kirstein and Fariñas, 2002; Glebova and Ginty, 2005; Lallemend and Ernfors, 2012). *In vitro* models using cultured sympathetic and dorsal root ganglia (DRGs) neurons that are maintained and then withdrawn from NGF have provided many key insights into the cell autonomous mechanisms that drive developmental neuronal cell death (Unsain et al., 2013, 2014; Geden et al., 2019). Recent work has shown that embryonic sensory neurons deprived of NGF results in PKC activation, ROS production, and TRPV1 activation which in turn induces a large increase in axoplasmic Ca^2+^ required for degeneration (Johnstone et al., 2018, 2019). To date, almost all studies have focused on NGF-sensitive peripheral neurons and mechanisms driving developmental neuronal death in other peripheral neuronal populations remains essentially unknown. In the present study, we asked whether the degenerative cascade initiated by NGF withdrawal could be extrapolated to population of neurons dependent on other neurotrophins, with a particular focus on the degenerative processes affecting BDNF-sensitive neurons.

Here, we show that NGF-dependent and BDNF-dependent DRG neurons undergo axonal blebbing, reduced axonal area, increased extracellular phosphatidylserine, and rise in intracellular Ca^2+^ when withdrawn from trophic support. Further, degeneration of both classes of neurons require the proapoptotic protein BAX. However, unlike NGF-sensitive neurons, degeneration of BDNF-dependent deprivation does not require Trk activity, PKC activity or caspase activity and instead requires reactive oxygen species (ROS). Together, these results highlight the complexity and divergence of the mechanisms underlying trophic factor deprivation-induced neuronal cell death during development in the PNS.

## Materials and Methods

### Mouse strains

CD1 mice were purchased from Charles River Laboratories. The previously described p75NTR knock-out mice (Lee et al., 1992) and BAX knock-out mice (Knudson et al., 1995) were maintained in a C57Bl6 strain background. Animal procedures and experiments were approved by the University of British Columbia animal care committee and the Canadian Council of Animal Care. Efforts were made to reduce animal handling and use.

### Culturing and trophic factor deprivation of DRG explants

DRGs were dissected from embryonic day (E)13.5 mouse embryos and seeded in 12-well plastic (Grenier) or four-well glass-bottom dishes (CellVis) sequentially coated with 1 mg/ml poly-d-lysine (Sigma-Aldrich), 10 μg/ml laminin-entactin complex (Corning), and 0.1 mg/ml PurCol bovine collagen (Advanced Biomatrix). Explants were grown in phenol-red Neurobasal media (Invitrogen) supplemented with 2% B27 serum-free supplement (Invitrogen), 1% l-glutamine (Wisent), 1% penicillin/streptomycin (Wisent), 10 μm 5-fluoro-2’-deoxyuridine (FDU; Sigma-Aldrich), and 12.5 ng/ml NGF (CedarLane) or 37.5 ng/ml BDNF (CedarLane) at 37°C, 5% CO_2_. Deprivation of neurotrophic support was accomplished using 2.0 μg/ml of function blocking antibodies against NGF (homemade rabbit polyclonal antibody raised against 2.5s NGF; Acheson et al., 1991) or BDNF (mouse monoclonal, DSHB #9-b) in complete fresh media without neurotrophic supplementation.

### βIII-tubulin immunocytochemistry, imaging and quantification of axon degeneration

DRG explants were fixed in 4% paraformaldehyde solution in PBS for 15 min, washed once in PBS and blocked in 5% milk in Tris-borate buffer and 0.3% Triton X-100 for 1 h at room temperature (RT). Explants were incubated overnight at 4°C with mouse monoclonal antibody against βIII-tubulin (Millipore, MAB5564) diluted 1:10,000 in blocking solution. DRGs were washed twice in PBS and then incubated with goat anti-mouse conjugated to Alexa Fluor 488 (Jackson ImmunoResearch, 115-545-003) diluted 1:5000 in blocking solution for a minimum of 3 h at RT. Explants were imaged using a Zeiss ObserverZ.1 inverted epifluorescence microscope with an automated motorized stage (5× magnification with tilling). From a stitched master image of the plate generated by Zen 2 software (Zeiss), quarter DRG fields were cropped to generate a set of images for analysis using the R script program Axoquant 2.0 (Johnstone et al., 2018). Final measurements were plotted as the mean axonal area of DRGs from three embryos. Increments of 500 μm were used for statistical analysis (normalized to same increments in control condition).

### Assessment of DRG explant survival with live Calcein-AM staining

DRG explants were treated with 1 μg/ml Calcein-AM (AAT Bioquest) in neurobasal media for 1 h at 37°C then switched to clear HBSS-based complete media supplemented with HEPES to maintain physiological pH. Explants were tiled-imaged using a Zeiss ObserverZ.1 inverted epifluorescence microscope with an automated motorized stage. From a stitched master image of the plate generated by the Zen 2 software, cell bodies and Schwann cells were cropped out and a binary mask image of each explants was created using NIH ImageJ software. Explant area and mean pixel intensity value corrected by the background signal were quantified to provide either the area of Calcein-AM-stained axons over a specified threshold or Calcein-AM fluorescence intensity per unit of area. DRG explants from the same embryo were pooled and averaged to generate the mean value for each embryo. Measurements were normalized relative to NGF or BDNF wild-type conditions.

### Annexin-V staining, imaging, and quantification

DRG explants seeded on glass bottom dishes (CellVis) were incubated with 1 μg/ml Annexin-V (AAT Bioquest) in annexin-V buffer (10 mm HEPES/NaOH, pH7.4, 140 mm NaCl, and 2.5 mm CaCl_2_) for 15 min at RT. DRGs were washed and tiled-imaged in the annexin-V buffer using a Zeiss Observer Z.1 inverted epifluorescence microscope (40× magnification). Stitched master images of each explant generated by Zen 2 software were cropped to eliminate soma and Schwann-cell area and axonal annexin-V area was measured using a binary mask over an established threshold for all explants. DRG explants from the same embryo were pooled and averaged to generate the mean value for each embryo. Measurements were normalized relative to NGF or BDNF controls.

### Ca^2+^ imaging with Fluo-4 and quantification

DRG explants were seeded on glass bottom dishes (CellVis) and treated with 5 μm Fluo-4 AM (Invitrogen) in neurobasal media for 15 min at 37°C, washed with HBSS and switched to clear HBSS-based complete media supplemented with HEPES (final concentration 20 mm) to maintain its physiological pH. Explants were tiled-imaged using a Zeiss ObserverZ.1 inverted epifluorescence microscope with an automated motorized stage at 40× magnification. Employing NIH ImageJ software, stitched master images of each explant were cropped to eliminated soma and Schwann-cell area. From there, a binary mask image of remaining axons was created to measure area and mean pixel intensity corrected by background signal. After calculating the intensity per unit of axonal area, DRG explants from the same embryo were pooled and averaged to generate the mean value per embryo. Measurements were normalized and expressed as fold-change from NGF or BDNF controls.

### Immunoblotting

For SDS-PAGE and Western blot analysis, a total of 25 DRG explants per well were seeded in 12-well plastic plates (Grenier). For protein harvesting, cultures were washed with PBS, and DRGs were scraped into 90 μl of sample buffer (4% SDS, 20% glycerol, 10% 2-mercaptoethanol, 0.004% bromophenol blue, and 0.125 m Tris-HCl, pH ∼6.8). Samples were boiled for 5 min, centrifuged, and stored at −80°C for later analysis. Antibodies used for immunoblotting were: anti-βIII-tubulin (Millipore MAB5564, 1:10,000), anti-neurofilament M (Millipore AB1987, 1:1000), anti-caspase-3 (NEB 9662, 1:1000), anti-TrkA (Millipore 06-574, 1:1000), anti-TrkB (Millipore 07-225, 1:1000), anti-TrkC (Millipore 07-226, 1:1000), and the previously described anti-p75NTR (Barker and Shooter, 1994).

### Pharmacological PKC, Trk, caspase, autophagy, translation, and necroptosis inhibitors

Stocks of PKC inhibitor Gö6976 (10 mm, Tocris 2253), Trk receptor inhibitor K252a (200 μm, Calbiochem #420298), pan-caspase inhibitors Boc-D-fmk (10 mm, Abcam ab142036), zVAD-fmk (20 mm, R&D Systems FMK001), and necroptosis inhibitor necrostatin-1 (NEC-1; 100 mm, Sigma-Aldrich N9037) were prepared in dimethylsulfoxide (DMSO) and used at 1:1000 dilution (final concentration of DMSO below 0.1%). The translation inhibitor cycloheximide (CHX; R&D Systems 0970/100) was dissolved at 1.0 g/l in water and used at 1:1000. Autophagy inhibitor 3-methyladenine (3-MA; Sigma-Aldrich M9281) was dissolved at 10 mm in phenol-red neurobasal media. Drugs were applied at the same time that the trophic factor withdrawal was initiated.

### EGTA, NAC, and NAD+ preparation

EGTA (AlfaAesar A16086, final concentration 5 mm), *N*-acetylcysteine (NAC; Sigma, A9165, final concentration 20 mm), or nicotinamide adenine dinucleotide (NAD+, Sigma-Aldrich, N7004, final concentration 5 mm) was dissolved in Neurobasal media, pH adjusted to 7.4, and filtered by 0.22 μm for final treatment of DRG explants. After 48 h of growth in NGF or BDNF, cultures were either maintained with trophic support or deprived of it, in the absence or presence of each specific compound for the entire deprivation period.

### Experimental design and statistical analysis

Data were plotted and analyzed using Prism 6 (GraphPad). All data were presented as mean ±SEM. The number of embryos *n* in each experiment or condition is described in each figure legend. Mann–Whitney test (unpaired, two-tailed) was used for two-group experiments comparisons. Two-way ANOVA with Bonferroni’s *post hoc* test or Tukey’s *post hoc* test was used to analyze differences in multiple groups. In all graphs, non-significant (*p* > 0.05): ns, * (or other symbols) *p* < 0.05, **p < 0.01, ***p < 0.001, and ****p < 0.0001.

## Results

**Figure 1.**
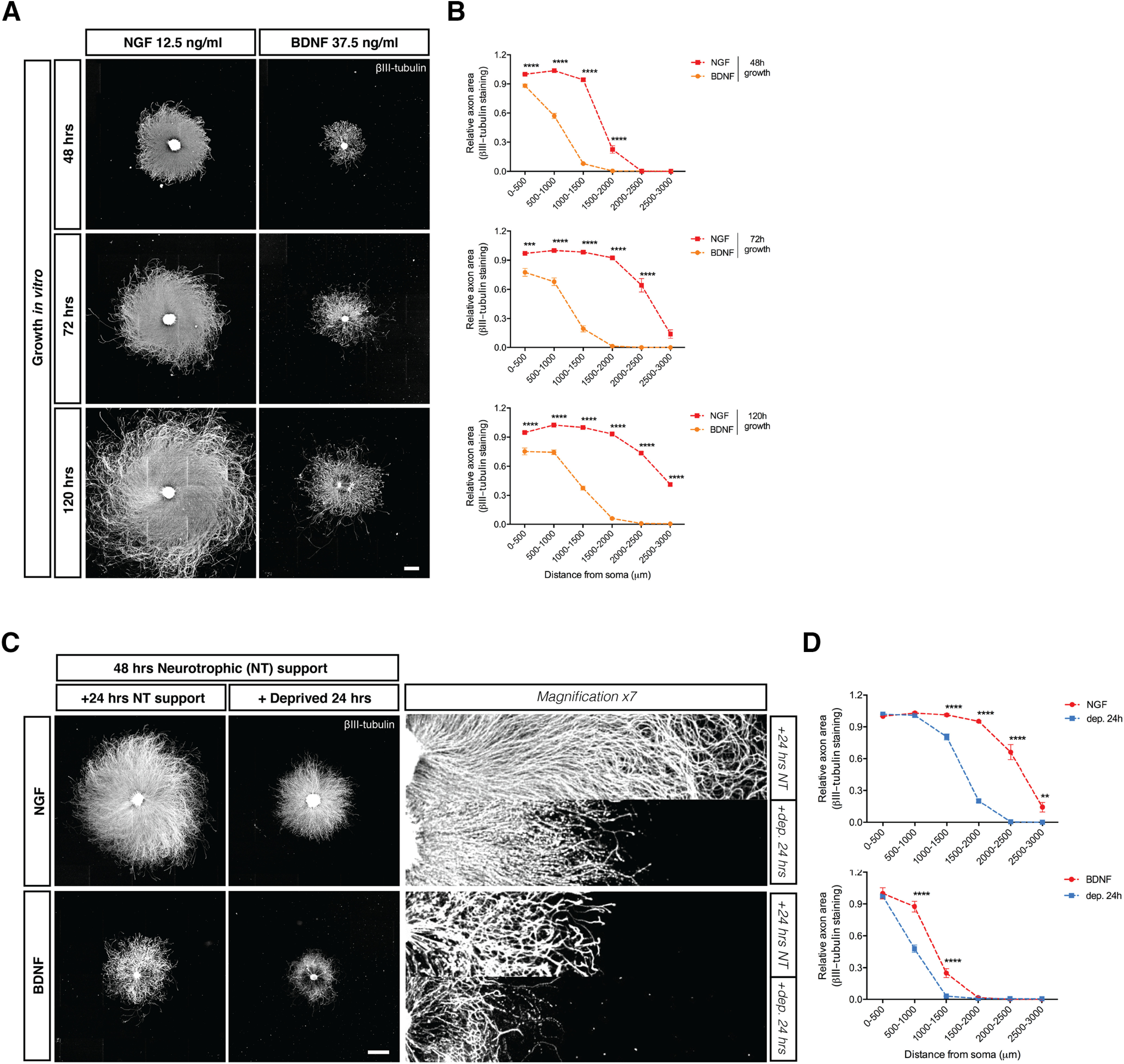
Comparative growth of NGF-dependent and BDNF-dependent DRG sensory neurons and their degeneration induced by trophic factor withdrawal. ***A***, βIII-tubulin staining of embryonic mice DRG explants cultured in the presence of NGF (12.5 ng/ml) or BDNF (37.5 ng/ml) for 48, 72, or 120 h. Scale bar: 1000 μm. ***B***, Quantification of axonal area as a function of the distance from the soma using Axoquant 2.0 (Johnstone et al., 2018) and plotted in 500-μm bins. The difference between the relative axonal area between NGF-dependent and BDNF-dependent DRG growth at different time points were analyzed by two-factor ANOVA and Bonferroni’s *post hoc* comparison and plotted with mean and SEM (*n* = 3 embryos for each condition; data shown are representative of three independent experiments); *NGF versus BDNF; ***p < 0.001, ****p < 0.0001. ***C***, DRG explants cultured in the presence of NGF or BDNF for 48 h and then either maintained with trophic support or deprived with a function blocking anti-NGF (2 μg/ml) or anti-BDNF (2 μg/ml) for the following 24 h, before fixation and immunostaining with βIII-tubulin. Scale bar: 1000 μm. ***D***, NGF and BDNF deprivation for 24 h results in a significant loss of βIII-tubulin-stained axons expressed as axonal area relative to 0–500 μm NGF or BDNF controls; analyzed by two-factor ANOVA and Bonferroni’s *post hoc* comparison and plotted with mean and SEM; **p < 0.01, ***p < 0.001, ****p < 0.0001.

**Figure 2.**
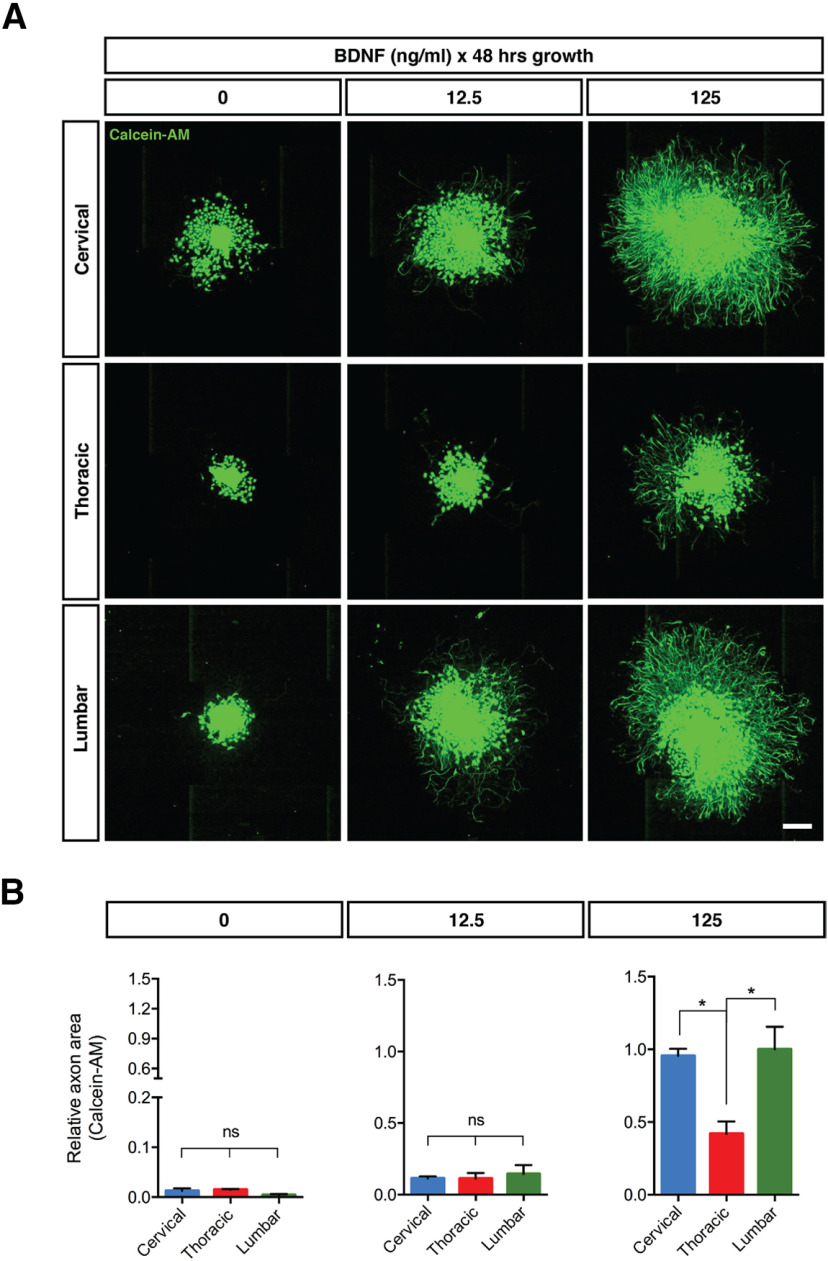
Axon growth of BDNF-dependent DRGs from the cervical, the thoracic or the lumbar region of the spinal cord with several concentration of BDNF. ***A***, Calcein-AM-stained DRGs from cervical, thoracic or lumbar spinal cord segments of E13.5 mice embryos were grown for 48 h in 0, 12.5, or 125 ng/ml of BDNF. Scale bar: 500 μm. ***B***, Quantification of Calcein-AM-stained axonal area relative to Calcein-AM-stained axonal area of lumbar DRGs at 125 ng/ml analyzed by one-factor ANOVA and Tukey’s *post hoc* comparison and plotted with mean and SEM (*n* = 3 embryos for each condition; data shown are representative of three independent experiments). ns: non-significant; *p < 0.05.

The apoptotic machinery involved in NGF deprivation-induced axonal degeneration in DRG neurons is well characterized (Geden et al., 2019). However, our knowledge of axonal degeneration induced by BDNF deprivation is rudimentary. To begin to address this, we characterized BDNF withdrawal-induced axon degeneration in DRG neurons generated from E13.5 mice embryos. Figure 1*A* shows that E13.5 DRGs cultured in the presence of BDNF survived and developed neurites (quantified in Fig. 1*B*). The extent and density of neurites was maximal at a BDNF concentration of 125 ng/ml (Fig. 2*A*), but even at this concentration, processes were significantly less dense and shorter than within parallel DRGs cultured in NGF (data not shown). It was also noted that DRGs derived from the lumbar and cervical parts of the spinal cord extended more exuberant processes in response to BDNF than DRGs derived from the thoracic region (Fig. 2*B*). For subsequent experiments, cervical DRG neurons were routinely cultured using 37.5 ng/ml of BDNF or 12.5 ng/ml of NGF. For BDNF-deprivation studies, cells were grown in BDNF for 48 h and then switched to BDNF-free media supplemented with an anti-BDNF monoclonal antibody for 24 h. Axons maintained and then deprived of BDNF in this manner showed morphologic signs of degeneration and blebbing (Fig. 1*C*, higher magnification, quantified in *D*).

**Figure 3.**
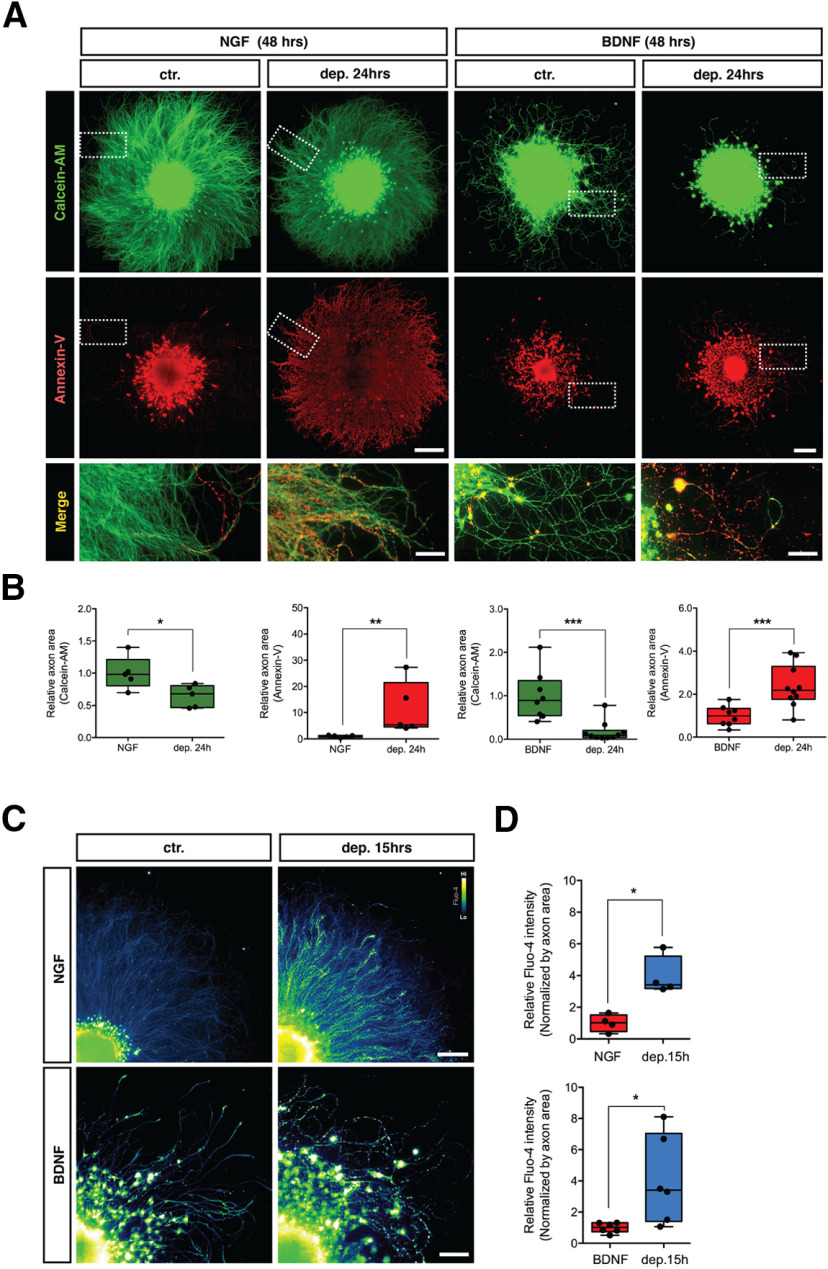
DRG sensory neurons undergoing BDNF deprivation display increased extracellular phosphatidylserine and increased axoplasmic Ca^2+^. ***A***, DRG explants cultured in the presence of NGF or BDNF for 48 h and then either maintained with trophic support (ctr) or deprived with an antibody against NGF or BDNF for the following 24 h (dep) were co-stained with Calcein-AM (green) and Annexin-V (red) to measure the area of healthy axons versus axons displaying phosphatidylserine, an apoptotic marker, respectively. NGF scale bar: 1000 μm and merge scale bar: 50 μm; BDNF scale bar: 500 μm and merge scale bar: 50 μm. ***B***, Both NGF and BDNF deprivation induced a significant decrease in Calcein-AM-positive axonal area (*n* = 5 embryos in NGF and *n* = 8 embryos in BDNF from pooled litters) and a significant increase in Annexin-V area (*n* = 5 embryos in NGF deprivation 24 h and *n* = 10 embryos in BDNF deprivation 24 h from pooled litters). The bar plots show mean, min/max, and 25/75% for each panel, analyzed by two-tailed Mann–Whitney tests with *p < 0.05, **p < 0.01, ***p < 0.001. ***C***, DRG explants cultured in NGF or BDNF were maintained in trophic media (ctr) or withdrawn from trophic support for 15 h (dep) before staining with Fluo-4 and imaged by epifluorescence microscopy. NGF scale bar: 1000 μm; BDNF scale bar: 200 μm. ***D***, Both NGF and BDNF deprivation induced a significant increase in axonal Fluo-4 intensity (*n* = 4 embryos in NGF and *n* = 6 embryos in BDNF from pooled litters). The Box plots show mean, min/max, and 25/75% for each panel, analyzed by two-tailed Mann–Whitney tests with *p < 0.05.

Cell biological and biochemical indications of BDNF-withdrawal induced axonal degeneration were also established. DRG axons that were maintained and then deprived of either NGF or BDNF show a significant increase of extracellular phosphatidylserine, determined using Annexin-V staining, and a drastic decrease of viable axons, determined using Calcein-AM (Fig. 3*A*, quantified in *B*). It has been previously shown that NGF deprivation induces a large increase in axoplasmic Ca^2+^ ∼15 h after deprivation (Johnstone et al., 2018, 2019) and here show that BDNF-withdrawal induces a similar elevation in axonal Ca^2+^ 15 h after trophic deprivation (Fig. 3*C*, quantified in *D*).

**Figure 4.**
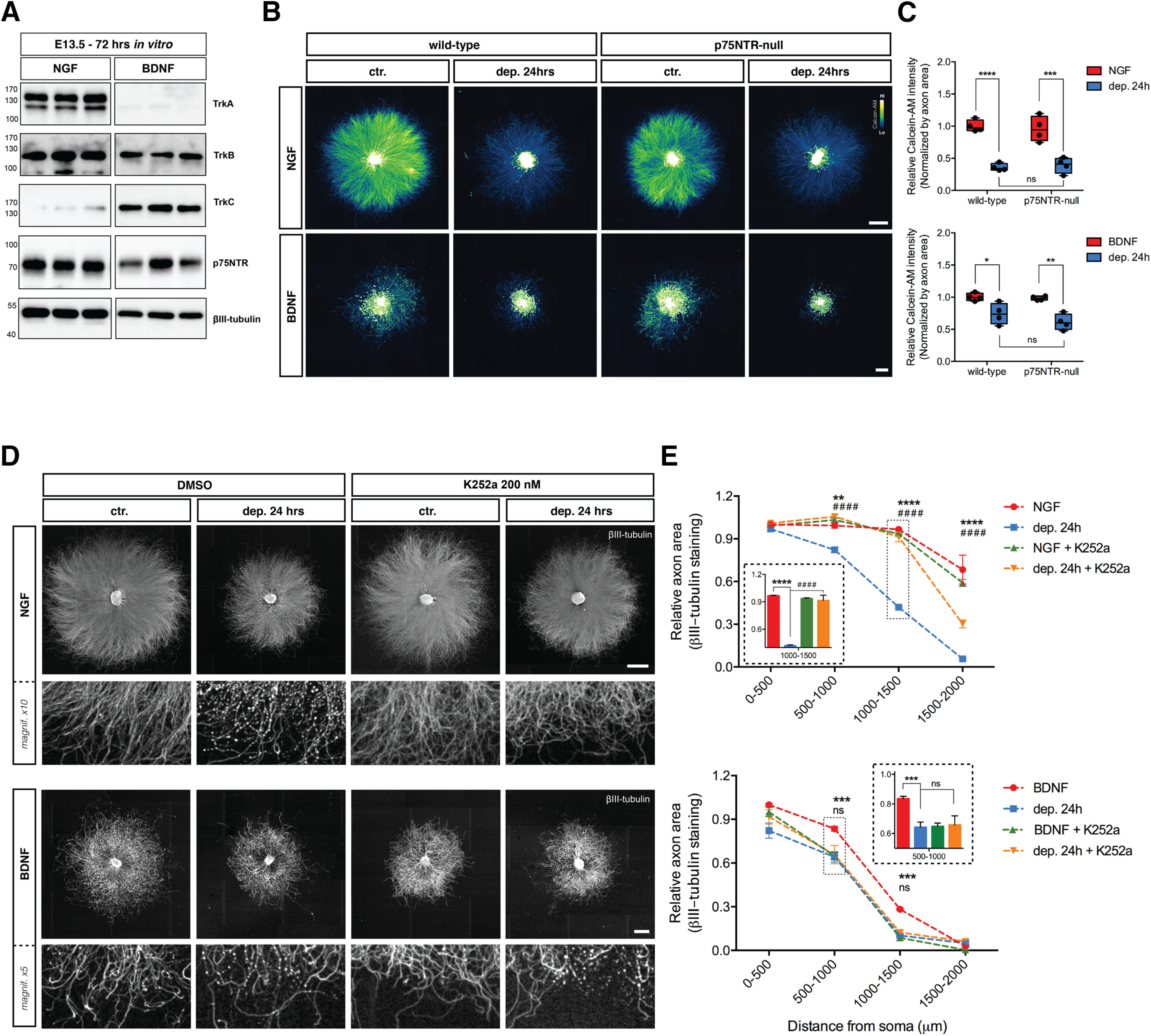
Trk receptor inhibition protects axons from NGF deprivation but not from BDNF deprivation, whereas p75NTR deficiency confers no protection to axons established in NGF or BDNF. ***A***, Protein lysates collected from E13.5 DRG explants cultured in the presence of NGF (12.5 ng/ml) or BDNF (37.5 ng/ml) for 48 h were analyzed by immunoblot against TrkA, TrkB, TrkC, p75NTR, and βIII-tubulin. ***B***, p75NTR knock-out does not rescue axons from degeneration after NGF or BDNF withdrawal. DRG explants from mixed-genotyped E13.5 litters were cultured in the presence of NGF or BDNF for 48 h and then either maintained or withdrawal from trophic support for 24 h before being lived stained with Calcein-AM. NGF scale bar: 1000 μm; BDNF scale bar: 500 μm. ***C***, Quantification of Calcein-AM intensity normalized by axonal area and relative to wild-type control. Non-significant difference was observed between wild-type and p75NTR-null DRG explants deprived of NGF or BDNF (*n* = 4 embryos in NGF and *n* = 4 embryos in BDNF from pooled litters). Analyzed by two-way ANOVA and Tukey’s *post hoc* comparison and plotted with median and SEM; *p < 0.05, **p < 0.01, ***p < 0.001, ****p < 0.0001. ***D***, DRG explants cultured in NGF or BDNF were either maintained in trophic media or withdrawn from trophic support with or without the Trk inhibitor K252a (200 nm) for 24 h before fixing, immunostaining for βIII-tubulin, and imaged by epifluorescence microscopy. NGF scale bar: 1000 μm; BDNF scale bar: 500 μm. ***E***, K252a rescued degeneration induced by NGF deprivation but not by BDNF deprivation. Quantification of axonal area as a function of the distance from the soma using Axoquant 2.0 and plotted in 500-μm binned segments relative to 0–500 μm NGF control (upper panel) or BDNF control (lower panel). The relative axonal area was analyzed by two-factor ANOVA followed by Tukey’s *post hoc* comparison and plotted with mean and SEM (*n* = 3 embryos per condition for each condition; data shown are representative of three independent experiments); *ctr. versus dep. 24 h; #dep. 24 h versus dep. 24 h + K252a; ns: non-significant, **p < 0.01, ***p < 0.001, ****p < 0.0001.

To characterize the neurotrophin receptor complement in DRG explants, protein lysates from E13.5 DRGs maintained in NGF or BDNF for 72 h were analyzed by immunoblot. DRGs cultured in NGF expressed abundant TrkA, TrkB, and p75NTR but low amounts of TrkC. In contrast, DRG neurons cultured in BDNF expressed abundant TrkB, TrkC, and p75NTR (Fig. 4*A*) but essentially no TrkA.

Previous studies have indicated that p75NTR is required for cell death of sympathetic neurons during development (Deppmann et al., 2008) but not for apoptosis of DRG sensory neurons. p75NTR has also been shown to be required for sympathetic neuron axon degeneration (Bamji et al., 1998; Singh et al., 2008). To determine whether p75NTR is required for axonal loss after NGF or BDNF deprivation in DRG axons, we assessed axonal loss in DRGs from p75NTR-null embryos (Fig. 4*B*). When maintained and then withdrawn from NGF or BDNF, the degree of axonal degeneration was the same in wild-type and p75NTR-null DRGs (Fig. 4*C*), ruling out a direct role for p75NTR in axon loss induced by neurotrophin withdrawal.

TrkA and TrkC have been implicated as dependence receptors (Nikoletopoulou et al., 2010), and recent studies have suggested that NGF deprivation activates a TrkA-dependent apoptotic signaling pathway (Feinberg et al., 2017). Consistent with this, Figure 4*D*,*E* shows that a low concentration of the pan-Trk inhibitor K252a (200 nm) rescues NGF deprivation induced axon degeneration of DRG sensory neurons but has no effect on BDNF deprivation-induced DRG axon degeneration (Fig. 4*D*, quantified in *E*). These results are consistent with previous findings showing that TrkB does not have dependence receptor activity (Nikoletopoulou et al., 2010).

**Figure 5.**
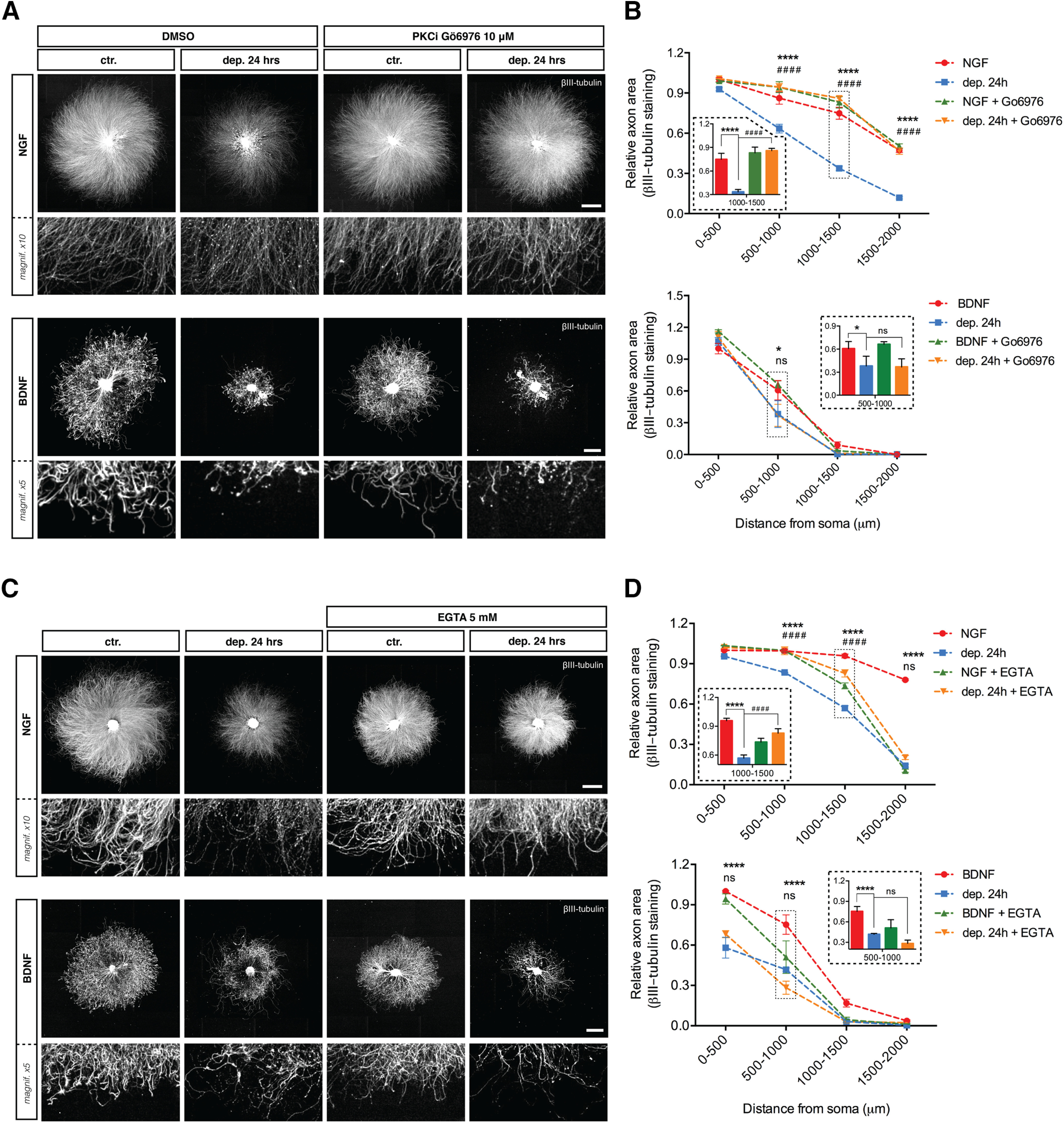
PKC inhibitor Gö6976 and EGTA rescue degeneration induced by NGF deprivation but not BDNF deprivation. ***A***, DRG explants cultured in NGF or BDNF were either maintained in trophic media or withdrawn from trophic support with or without PKC inhibitor Gö6976 (10 μm) for 24 h before fixing, immunostaining for βIII-tubulin, and imaged by epifluorescence microscopy. NGF scale bar: 1000 μm; BDNF scale bar: 500 μm. ***B***, Quantification of axonal area as a function of the distance from the soma using Axoquant 2.0 (Johnstone et al., 2018) and plotted in 500-μm bins segments relative to 0–500 μm 48-h time point. The relative axonal area was analyzed by two-factor ANOVA followed by Tukey’s *post hoc* comparison and plotted with mean and SEM (*n* = 3 embryos per condition for each condition; data shown are representative of three independent experiments); *control versus deprived 24 h; #deprived 24 h versus deprived 24 h + Gö6976; ns: non-significant, *p < 0.05, ****p < 0.0001. ***C***, DRG explants cultured in NGF or BDNF were either maintained in trophic media or withdrawn from trophic support with or without Ca^2+^ chelator EGTA (5 mm) for 24 h before fixing, immunostained for βIII-tubulin, and imaged by epifluorescence microscopy. NGF scale bar: 1000 μm; BDNF scale bar: 500 μm. ***D***, Quantification of axonal area as a function of the distance from the soma using Axoquant 2.0 and plotted in 500-μm binned segments. Ca^2+^ chelation rescued degeneration induced by NGF deprivation but not by BDNF deprivation. The relative axonal area was analyzed by two-factor ANOVA followed by Tukey’s *post hoc* comparison and plotted with mean and SEM (*n* = 3 embryos per condition for each condition; data shown are representative of three independent experiments); *ctr. versus dep. 24 h; #dep. 24 h versus dep. 24 h + EGTA; ns: non-significant, ****p < 0.0001.

To begin to discern signaling mechanisms driving BDNF deprivation-induced axon loss, we tested several compounds known to inhibit NGF withdrawal-induced axon degeneration or to inhibit neuronal cell death. PKC inhibitor Gö6976 rescues NGF deprivation-induced apoptosis (Johnstone et al., 2019) but had no effect on BDNF deprivation (Fig. 5*A*, quantified in *B*). Likewise, the Ca^2+^ chelator EGTA is a potent inhibitor of axon loss induced by NGF withdrawal in DRG neurons (Johnstone et al., 2018) but did not protect against BDNF deprivation (Fig. 5*C*, quantified in *D*). The translation inhibitor CHX also significantly protects axons from degeneration induced by NGF deprivation (Fig. 6*A*) but has no effect on axon degeneration induced by BDNF withdrawal. Finally, neither the autophagy inhibitor 3-MA, the necroptosis inhibitor NEC-1 nor NAD+ blocked BDNF withdrawal-induced axonal degeneration of DRG sensory neurons (Fig. 6*B–D*).

**Figure 6.**
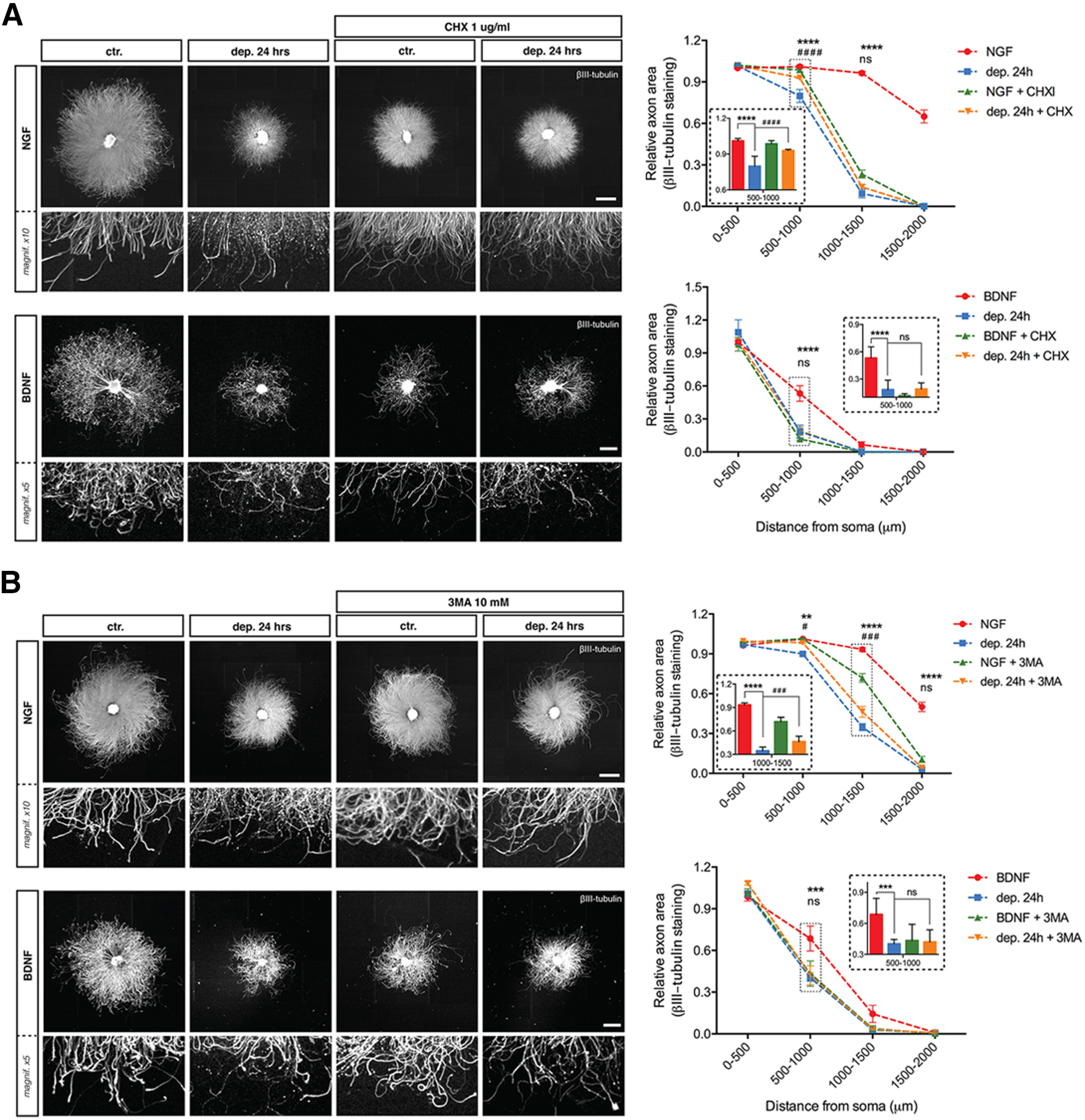
Translation, autophagy, necroptosis or Wallerian-like degeneration are not involved in BDNF deprivation-induced degeneration. ***A***, DRG explants cultured in NGF or BDNF were maintained in trophic media or withdrawn from trophic support with or without translation inhibitor CHX (1 μg/ml) for 24 h before fixing, immunostaining for βIII-tubulin, and imaging by epifluorescence microscopy. NGF scale bar: 1000 μm; BDNF scale bar: 500 μm. Quantification of axonal area as a function of the distance from the soma plotted in 500-μm bins segments relative to 0–500 μm BDNF control. CHX rescued degeneration induced by NGF deprivation but not by BDNF deprivation. The relative axonal area was analyzed by two-factor ANOVA followed by Tukey’s *post hoc* comparison and plotted with mean and SEM (*n* = 3 embryos per condition for each condition; data shown are representative of three independent experiments); *ctr. versus dep. 24 h; #dep. 24 h versus dep. 24 h + CHX; ns: non-significant, ****p < 0.0001. ***B***, DRG explants were withdrawn from trophic support with or without the autophagy inhibitor 3-MA (10 mm) for 24 h before being immunostained for βIII-tubulin. NGF scale bar: 1000 μm; BDNF scale bar: 500 μm. Quantification of axonal area as a function of the distance from the soma plotted in 500-μm bins segments relative to 0–500 μm BDNF control. 3-MA rescued degeneration induced by NGF deprivation but not by BDNF deprivation. The relative axonal area was analyzed by two-factor ANOVA followed by Tukey’s *post hoc* comparison and plotted with mean and SEM (*n* = 3 embryos per condition for each condition; data shown are representative of three independent experiments); *ctr. versus dep. 24 h; #dep. 24 h versus dep. 24 h + 3-MA; ns: non-significant, *p < 0.05, **p < 0.01, ***p < 0.001, ****p < 0.0001.

**Figure 6(Continued).**
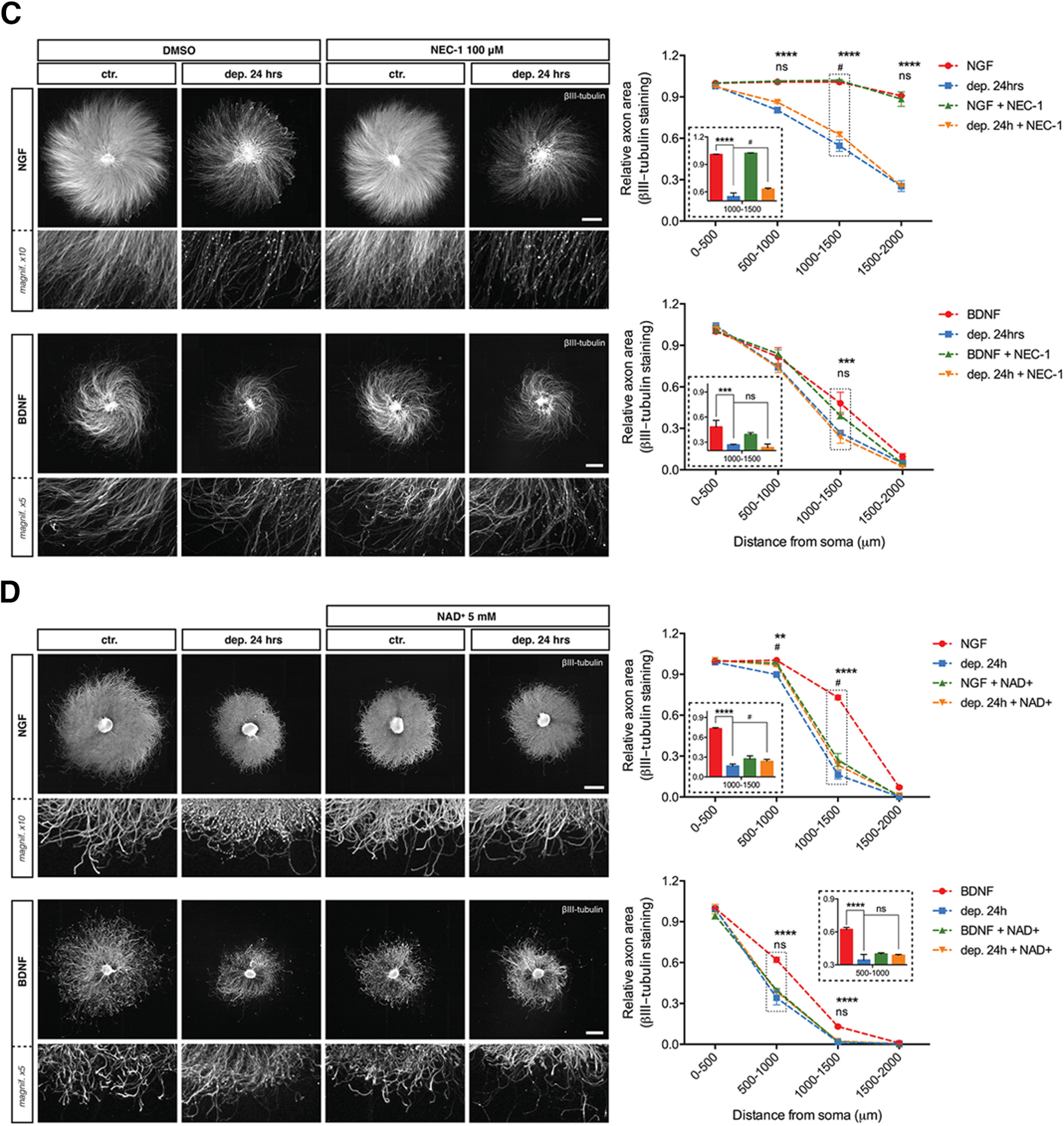
***C***, DRG explants were withdrawn from trophic support with or without the necroptosis inhibitor NEC-1 (100 μm) for 24 h before being immunostained for βIII-tubulin. NGF scale bar: 1000 μm; BDNF scale bar: 500 μm. Quantification of axonal area as a function of the distance from the soma plotted in 500-μm binned segments relative to 0–500 μm BDNF control. NEC-1 slightly rescued degeneration induced by NGF deprivation but not by BDNF deprivation. The relative axonal area was analyzed by two-factor ANOVA followed by Tukey’s *post hoc* comparison and plotted with mean and SEM (*n* = 3 embryos per condition for each condition; representative of three independent experiments); *ctr. versus dep. 24 h; #dep. 24 h versus dep. 24 h + NEC-1; ns: non-significant, *p < 0.05, ***p < 0.001, ****p < 0.0001. ***D***, DRG explants were withdrawn from trophic support with or without NAD+ (5 mm) for 24 h before being immunostained for βIII-tubulin. NGF scale bar: 1000 μm; BDNF scale bar: 500 μm. Quantification of axonal area as a function of the distance from the soma plotted in 500-μm bins segments relative to 0–500 μm BDNF control. NAD+ rescued degeneration induced by NGF deprivation but not by BDNF deprivation. The relative axonal area was analyzed by two-factor ANOVA followed by Tukey’s *post hoc* comparison and plotted with mean and SEM (*n* = 3 embryos per condition for each condition; data shown are representative of three independent experiments); *ctr. versus dep. 24 h; #dep. 24 h versus dep. 24 h + NAD+; ns: non-significant, *p < 0.05, **p < 0.01, ****p < 0.0001.

**Figure 7.**
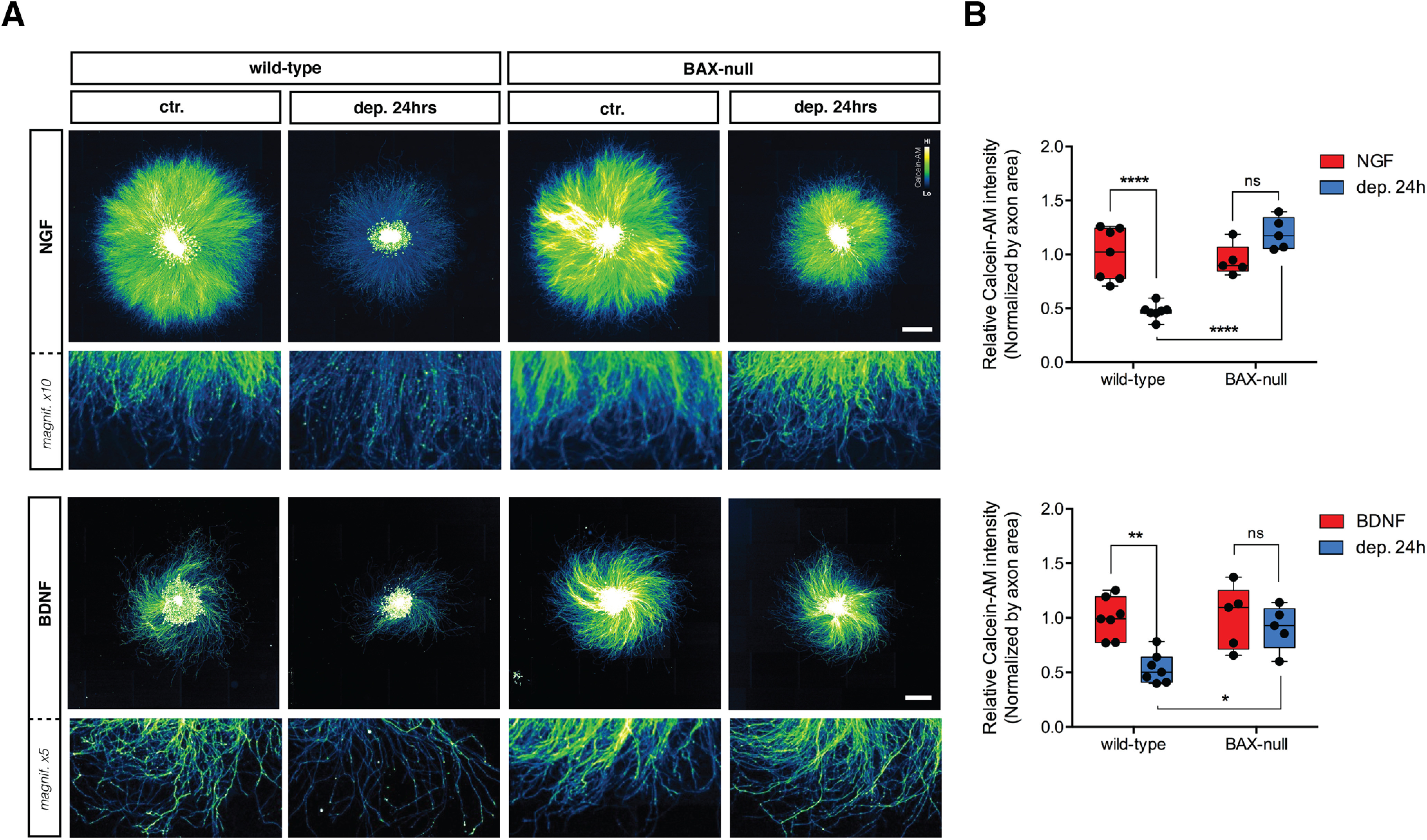
NGF and BDNF deprivation-induced degeneration require BAX. ***A***, DRG explants from mixed-genotyped E13.5 litters were cultured in the presence of NGF or BDNF for 48 h and then either maintained or withdrawn from trophic support for 24 h before being lived stained with Calcein-AM. NGF scale bar: 1000 μm; BDNF scale bar: 500 μm. ***B***, Quantification of Calcein-AM intensity normalized by axonal area and relative to wild-type control. A significant increase in Calcein-AM intensity was observed in both NGF or BDNF deprived BAX-null DRG explants compared with their deprived wild-type counterparts (*n* = 7 embryos in NGF/BDNF ctr., *n* = 5 embryos in NGF/BDNF dep. 24 h, from pooled litters). Data were analyzed by two-way ANOVA and Tukey’s *post hoc* comparison and plotted with median and SEM ns: non-significant, *p < 0.05, **p < 0.01, ****p < 0.0001.

BAX is a central player in neuronal apoptosis and crucial for NGF deprivation-induced axonal degeneration (Patel et al., 2000; Schoenmann et al., 2010; Simon et al., 2012). To address the role of BAX in BDNF-deprived DRG sensory neurons, BAX-null DRG neurons were maintained in NGF or BDNF and then deprived of trophic support. Figure 7 shows that axons lacking BAX were significantly protected from degeneration induced by NGF and BDNF deprivation (Fig. 7*A*, quantified in *B*).

**Figure 8.**
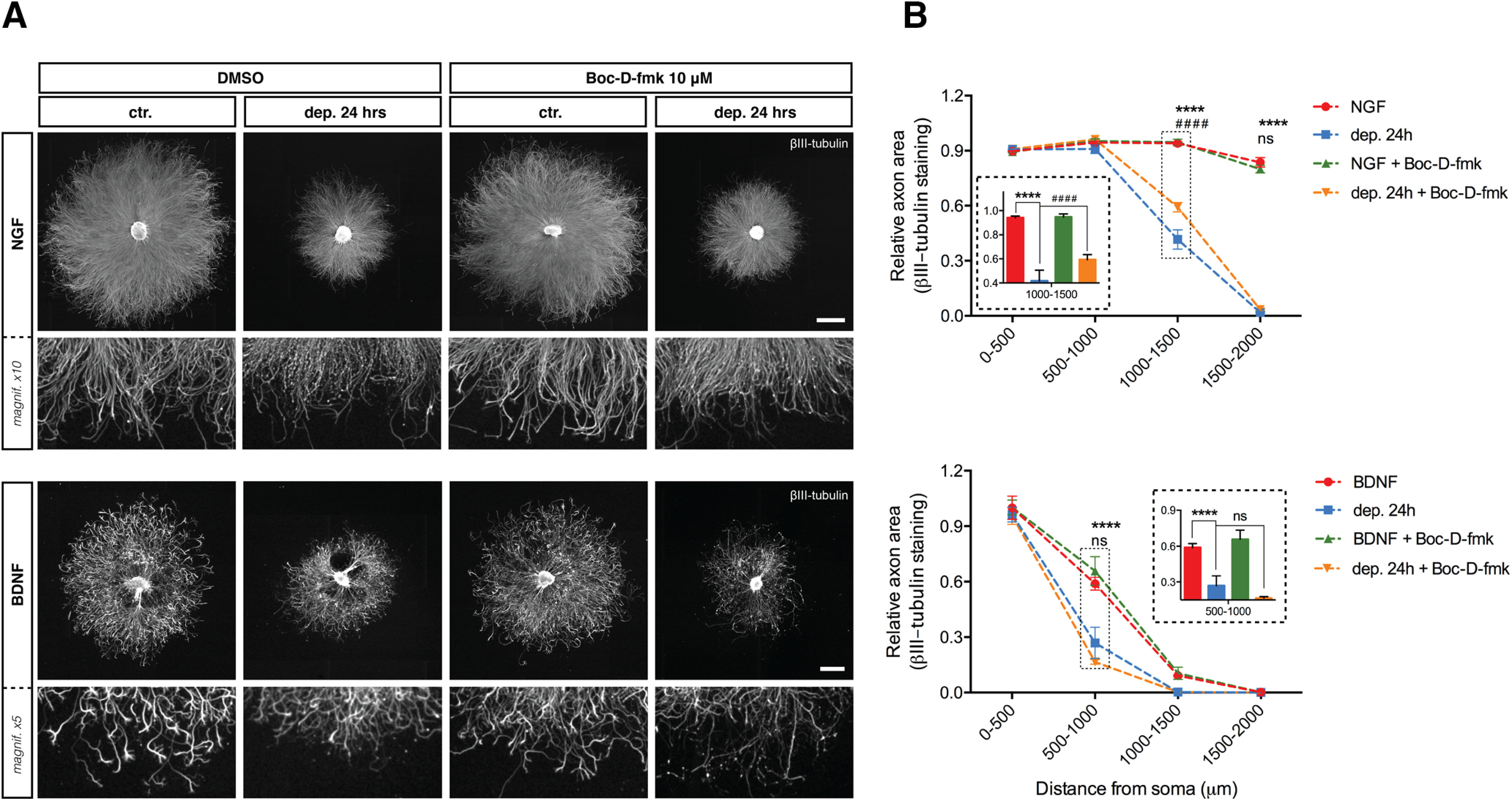
pan-Caspase inhibition does not block degeneration induced by BDNF deprivation. ***A***, DRG explants cultured in NGF or BDNF were maintained in trophic media or were withdrawn from trophic support with or without pan-caspase inhibitor Boc-D-fmk (10 μm) for 24 h before fixing, immunostaining for βIII-tubulin, and imaging by epifluorescence microscopy. NGF scale bar: 1000 μm; BDNF scale bar: 500 μm. ***B***, Quantification of axonal area as a function of the distance from the soma was performed using Axoquant 2.0 (Johnstone et al., 2018) and plotted in 500-μm binned segments relative to 0–500 μm NGF/BDNF controls. Pan-caspase inhibitor Boc-D-fmk rescued degeneration induced by NGF deprivation but not by BDNF deprivation. The relative axonal area was analyzed by two-factor ANOVA followed by Tukey’s *post hoc* comparison and plotted with mean and SEM (*n* = 3 for each condition; data shown are representative of three independent experiments); *ctr. versus dep. 24 h; #dep. 24 h versus dep. 24 h + Boc-D-fmk; ns: non-significant, ****p < 0.0001.

**Figure 9.**
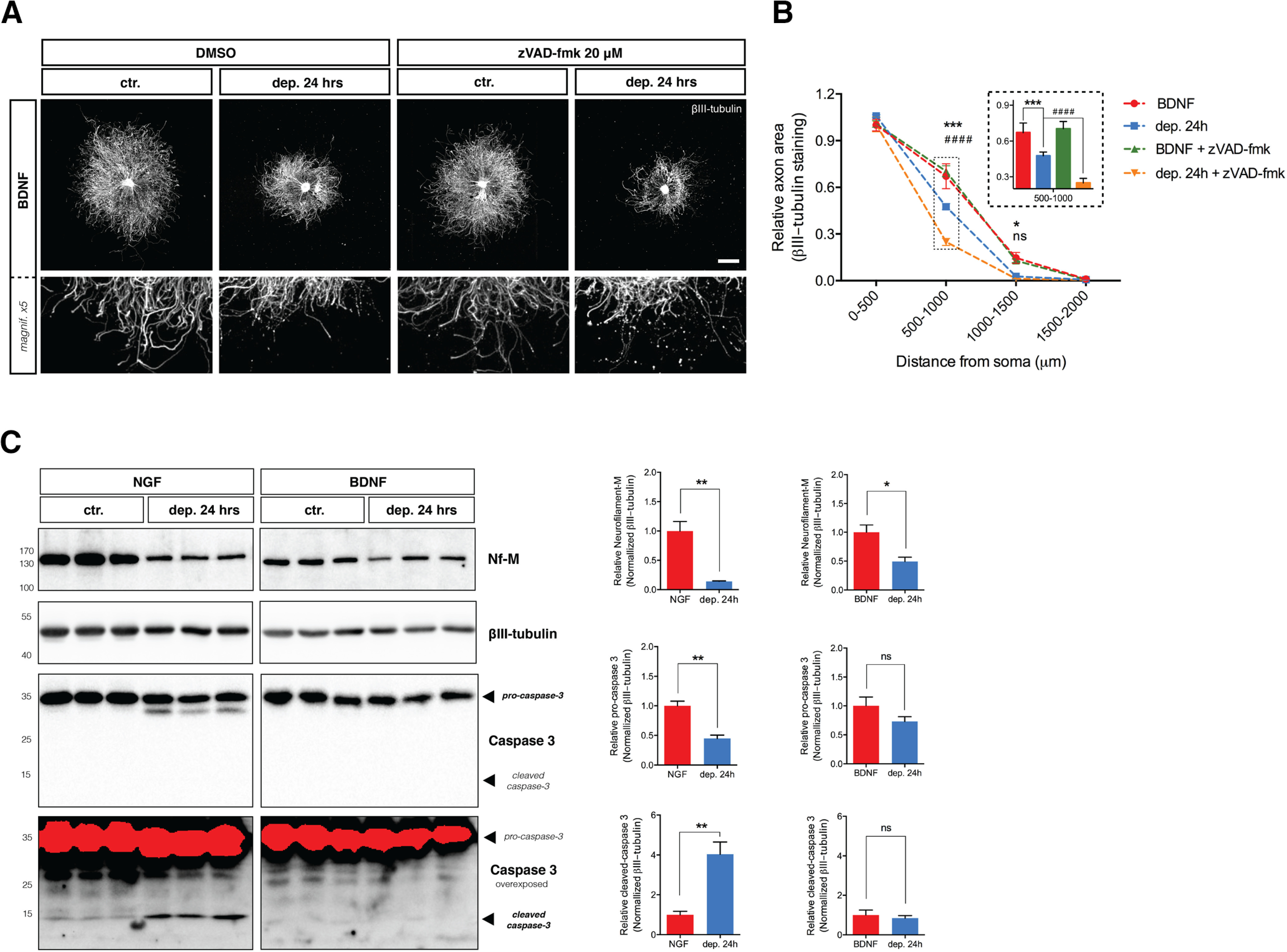
Cleaved form of executioner caspase-3 does not increase during BDNF deprivation. ***A***, DRG explants cultured in NGF or BDNF were either maintained in trophic media or withdrawn from trophic support with or without pan-caspase inhibitor zVAD-fmk (20 μm) for 24 h before fixing, immunostaining for βIII-tubulin, and imaged by epifluorescence microscopy. BDNF scale bar: 1000 μm. ***B***, Quantification of axonal area as a function of the distance from the soma plotted in 500-μm bins segments relative to 0–500 μm BDNF control. Pan-caspase inhibitor zVAD-fmk does not rescues degeneration induced by BDNF deprivation. The relative axonal area was analyzed by two-factor ANOVA followed by Tukey’s *post hoc* comparison and plotted with mean and SEM (*n* = 3 embryos per condition for each condition; data shown are representative of three independent experiments); *ctr. versus dep. 24 h; #dep. 24 h versus dep. 24 h + zVAD-fmk; ns: non-significant, *p < 0.05, ***p < 0.001, ****p < 0.0001. ***C***, Protein lysates collected from E13.5 DRG explants cultured in the presence of NGF (12.5 ng/ml) or BDNF (37.5 ng/ml) for 48 h were maintained or withdrawn from trophic support for 24 h and then analyzed by immunoblot against neurofilament-M (Nf-M) and caspase-3. Levels of Nf-M significantly deceased after either NGF and BDNF deprivation but only NGF deprived DRG lysates show a significant change in pro-caspase-3 and cleaved caspase-3 levels. Data were analyzed by two-tailed Mann–Whitney plotted with mean and SEM (*n* = 3 embryos per condition for each condition; representative of three independent experiments); ns: non-significant, *p < 0.05, **p < 0.01.

Caspase-3 is crucial for NGF deprivation-induced axonal degeneration (Simon et al., 2012; Unsain et al., 2013) and the requirement for BAX in BDNF withdrawal-induced axonal loss suggests that caspases may also play a role in axonal degeneration induced by BDNF deprivation. However, Figure 8*A* shows that while caspase inhibition efficiently rescued axons from NGF deprivation, two distinct pan-caspase inhibitors (Boc-D-fmk and zVAD-fmk) did not reduce axonal degeneration in neurons that were maintained and then withdrawn from BDNF (Figs. 8*A*, quantified in *B*, and 9*A*, quantified in *B*). Correspondingly, NGF deprivation decreased levels of pro-caspase-3 and increased cleaved caspase-3, whereas levels of pro-caspase-3 and cleaved caspase-3 did not change in neurons maintained and then withdrawn from BDNF for 15, 24 and 30 h (Fig. 9*C*; data not shown). Taken together, these results indicate that BAX activity mediates BDNF deprivation-induced axonal degeneration through a caspase-independent pathway.

**Figure 10.**
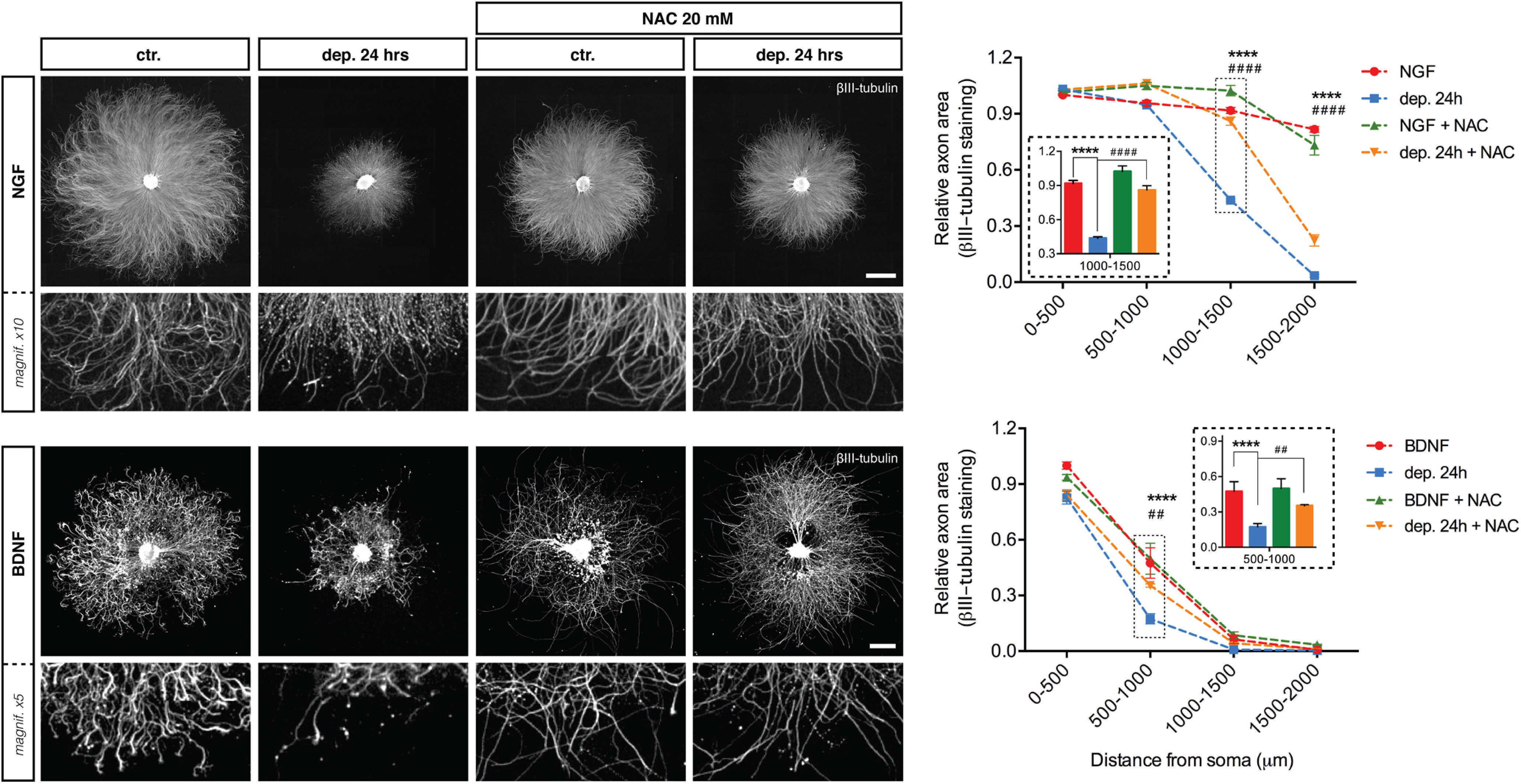
ROS are required for axon degeneration induced by BDNF deprivation. ***A***, DRG explants cultured in NGF or BDNF were either maintained in trophic media or withdrawn from trophic support with or without ROS scavenger NAC (20 mm) for 24 h before fixing, immunostaining for βIII-tubulin, and imaged by epifluorescence microscopy. NGF scale bar: 1000 μm; BDNF scale bar: 500 μm. ***B***, Quantification of axonal area as a function of the distance from the soma and plotted in 500-μm bins segments relative to 0–500 μm 48-h time point. NAC rescued degeneration induced by NGF deprivation and BDNF deprivation. The relative axonal area was analyzed by two-factor ANOVA followed by Tukey’s *post hoc* comparison and plotted with mean and SEM (*n* = 3 embryos for each condition; data shown are representative of three independent experiments); *ctr. versus dep. 24 h; #dep. 24 h versus dep. 24 h + NAC; **p < 0.01, ****p < 0.0001.

Several reports have shown that BAX can facilitate production of mitochondrial ROS (Kirkland and Franklin, 2001, 2007; Kirkland et al., 2002, 2010). To explore whether ROS play a role in neurotrophin deprivation-induced axonal degeneration, axons maintained in NGF or BDNF were exposed to NAC, a ROS scavenger, and then withdrawn from trophic support. Figure 10 shows that axonal degeneration induced by either NGF or BDNF deprivation was blocked in the presence of NAC, indicating that ROS are required for axonal degeneration induced by neurotrophin deprivation.

## Discussion

The mammalian PNS has proven a useful system for identifying specific mechanisms that are required for developmental neuronal degeneration. Substantial understanding of processes that mediate neuronal cell death and axonal destruction has been obtained from analyses of NGF-dependent DRG neurons maintained *in vitro*. However, less is known about signaling pathways that lead to the developmental loss of other sensory neuron populations. In this study, we have examined mechanisms that promote the developmental degeneration of BDNF-dependent sensory neurons. Our observations show that BDNF-dependent DRG sensory neurons employ destructive mechanisms distinct from those employed by NGF-dependent sensory neurons.

**Growth differences in NGF-dependent and BDNF-dependent DRG populations.** Several studies point to BDNF as a key trophic factor required to sustain the survival of different neuronal populations *in vivo* and *in vitro* (Johnson et al., 1986; Kalcheim et al., 1987; Liebl et al., 1997). Cranial sensory neurons are highly dependent on BDNF for survival and growth (Hellard et al., 2004), whereas only a subpopulation of DRG sensory neurons requires BDNF for survival during development (Huber et al., 2000; Valdés-Sánchez et al., 2010). Here, we showed that BDNF supports the survival and growth of neurons within E13.5 DRG explants, with neurite length steadily increasing with the time of trophic factor exposure. However, BDNF-dependent outgrowth was considerably less than that supported by NGF, consistent with the observation that only 8% of DRG neurons are TrkB+, while 80% are TrkA+ (Fariñas et al., 1998; Ernsberger, 2009). Thus, in the absence of NGF, the vast majority of DRG sensory neurons degenerate, leaving behind a small number of TrkB+ neurons. The reduced capacity of BDNF to promote neurite extension in culture may also reflect the fact that TrkB, but not TrkA, is downregulated after exposure and binding to its ligand (Sommerfeld et al., 2000; Haapasalo et al., 2002) and that BDNF activates Ras considerably less effectively than NGF (Borasio et al., 1989; Carter et al., 1995).

**Trophic deprivation-induced degeneration of BDNF-dependent DRG sensory neurons.** To mimic BDNF deprivation that occurs during embryonic development, E13.5 DRGs were maintained in BDNF and then withdrawn from the factor. A function blocking monoclonal antibody directed against BDNF was deployed to inactivate any residual BDNF remaining. BDNF deprivation resulted in neurite blebbing, a hallmark morphology of degenerating neurites, and caused a significant reduction of area occupied by neurites. Axonal degeneration provoked by BDNF deprivation was confirmed using the live dye Calcein-AM and by staining with Annexin-V, which detects phosphatidylserine on the outer leaflet of the plasma membrane, a prototypical signal driving phagocytosis of cells undergoing cell death (Wakatsuki and Araki, 2017; Shacham-Silverberg et al., 2018). Calcein-AM is a sensitive staining technique to quantify axonal integrity. However, Calcein-AM binds calcium after being hydrolyzed by intracellular esterase and its use was not compatible with some of our treatments (e.g., EGTA). Therefore, Calcein-AM staining was used to follow the effect of p75NTR or BAX deficiency on axonal integrity during trophic deprivation conditions and the effects of drugs on axonal degeneration was studied based on βIII-tubulin staining and quantified with Axoquant 2.0 (as described in Johnstone et al., 2018).

**How does BDNF deprivation trigger degeneration in BDNF-dependent sensory neurons?** Several studies have indicated that unliganded TrkA promotes proapoptotic signaling in sympathetic and sensory neurons withdrawn from NGF (Tauszig-Delamasure et al., 2007; Nikoletopoulou et al., 2010; Feinberg et al., 2017). In this sense, TrkA can be considered a “dependence receptor” that promotes survival signaling when bound by ligand but drives death signaling on ligand withdrawal (Nikoletopoulou et al., 2010). Here, we showed that the pan-Trk kinase inhibitor K252a prevented degeneration normally induced by NGF deprivation but had no effect on degeneration induced by BDNF deprivation, indicating that TrkA, but not TrkB, behaves as a dependence receptor. This finding agrees with those of Barde’s group who found that TrkA and TrkC behave as dependence receptors but the BDNF receptor TrkB is incapable of doing so (Nikoletopoulou et al., 2010).

We also questioned the role of p75NTR in BDNF deprivation. Depending on the cellular and molecular context, the low-affinity neurotrophin receptor can drive prosurvival or prodeath signaling (Roux and Barker, 2002; Mehlen and Bredesen, 2004). Although p75NTR is crucial for sympathetic neuronal remodeling during embryonic development (Bamji et al., 1998; Singh et al., 2008), here we found that p75NTR had no effect on degeneration of sensory neurons maintained and then withdrawn from either NGF or BDNF.

PKC plays an indispensable role in DRG degeneration induced by NGF withdrawal (Johnstone et al., 2019) but PKC inhibitors had no effect on BDNF-withdrawal induced degeneration. Likewise, CHX, a potent blocker of NGF withdrawal-induced degeneration had no effect on BDNF-withdrawal induced deprivation. Therefore, degeneration mechanisms of sensory neurons maintained and then withdrawn from BDNF are fundamentally distinct from those in NGF-dependent sensory neurons.

**Role of Ca^2+^ in BDNF deprivation induced degeneration of BDNF-dependent DRG sensory neurons.** In NGF-dependent DRG neurons, extracellular Ca^2+^ chelation blocks both the axoplasmic Ca^2+^ rise and the subsequent degenerative process that normally occur on NGF withdrawal (Johnstone et al., 2018, 2019). Here, we showed that BDNF deprivation induces Ca^2+^ rise in neurites of BDNF-dependent DRG explants yet Ca^2+^ chelation with EGTA did not rescue BDNF deprivation-induced degeneration. We observed that Ca^2+^ chelation in non-deprived DRG explants induced growth arrest and previous work has established that the ability of BDNF to sustain neuronal survival is reduced in comparison to NGF (Borasio et al., 1989; Carter et al., 1995). These results suggest a delicate homeostasis within BDNF-dependent DRG neurons. The lack of Ca^2+^ paired with the trophic support deprivation could, in these sensitive cells, favor degeneration instead of protection. Therefore, our results do not completely rule out an active role of Ca^2+^ in the degenerative mechanism of BDNF-deprived DRG neurons.

**ROS play a central role in the degeneration induced by BDNF deprivation.** ROS were initially described solely as toxic cellular by-products, but a growing body of evidence has established ROS as endogenous modulators of numerous physiological functions (Wilson et al., 2018). A recent study showed that NGF deprivation in sensory neurons induces ROS production through a PKC/NOX pathway and that ROS scavengers rescue degeneration of NGF-dependent sensory neurons after trophic deprivation (Johnstone et al., 2019). In the present work we showed that the antioxidant NAC partially protects DRG neurons from BDNF deprivation, suggesting that ROS play a role in the degeneration of BDNF-dependent sensory neurons. However, blocking PKC during BDNF deprivation had no effect on degeneration, indicating that the contribution of NOX-derived ROS to BDNF degeneration pathway is likely minor. Consistent with this, we found that NOX inhibitors that block NGF withdrawal-induced degeneration had no effect on BDNF-withdrawal-induced degeneration (data not shown).

Aside from NOX complexes, the other major source of ROS in the cell is mitochondria. Our results show that BAX is required for BDNF deprivation-induced degeneration of DRG neurons *in vitro*, consistent with *in vivo* data showing the importance of BAX during developmental cell death of BDNF-dependent cranial sensory neurons, particularly from nodose, petrosal and vestibular ganglia (Hellard et al., 2004). BAX translocates to the mitochondria and induces mitochondria outer membrane permeabilization (MOMP; Kalkavan and Green, 2018); in many circumstances MOMP provokes the release of the proapoptotic proteins SMAC and cytochrome *c*, engaging in the recruitment and activation of executioner caspases. However, since cleaved caspase-3 levels did not rise, and caspase blockers did not slow neuronal loss, in DRG sensory neurons deprived of BDNF, BAX must facilitate cell loss through a caspase-independent mechanism in this setting. BAX-dependent and caspase-independent cell death typically involves mitochondrial potential loss and failure (Deshmukh et al., 2000; Chang and Johnson, 2002; Chang et al., 2003; Lang-Rollin et al., 2003), with BAX-mediated MOMP inducing an increase of mitochondrial ROS production (Jiang et al., 2008; Garcia-Perez et al., 2012). In some circumstances, BAX-mediated MOMP and ROS production can trigger the formation of the mitochondria permeability transition pore which has been implicated in several forms of neuronal death (Lamarche et al., 2013).

A recent review by Fricker et al. (2018) proposed the existence of at least twelve different cell death pathways, highlighting the diversity and complexity of cellular death mechanisms (Fricker et al., 2018). Here, we examined prodegenerative pathways such as necroptosis and autophagy and mechanisms such as protein translation and NAD+ metabolism. Our results showed that several of these pathways impinge on the degenerative process induced by NGF deprivation but blockade of necroptosis, autophagy or translation nor NAD+ supplementation rescued degeneration evoked by BDNF withdrawal.

In conclusion, we have provided the first in depth characterization of the mechanisms that mediate degeneration of BDNF-dependent DRG sensory neurons on trophic factor withdrawal. We show that the pathways regulating the degeneration of BDNF-dependent DRG sensory neurons requires BAX and ROS but are Trk and caspase independent and distinct from those invoked on NGF withdrawal.

## Synthesis

Reviewing Editor: Francisca Bronfman, Universidad Andrés Bello Decisions are customarily a result of the Reviewing Editor and the peer reviewers coming together and discussing their recommendations until a consensus is reached. When revisions are invited, a fact-based synthesis statement explaining their decision and outlining what is needed to prepare a revision will be listed below. The following reviewer(s) agreed to reveal their identity: Yves-Alain Barde.

The authors have compared the pathways of degeneration of DRG neurons deprived of NGF or BDNF. They have

examined this in DRG explants and have utilized a variety of different inhibitors (as well as Bax and p75 KO neurons) to study the mechanism of axonal degeneration of these two distinct populations of neurons. The main result is that the mechanisms involved in the degeneration of the sub-population supported by either factor differ. This is an exciting and mostly novel conclusion that is well worth reporting.

The primary concern the reviewers agree upon was the assay to measure axonal degeneration. The assay relay meanly in the determination of axonal area covered by beta-tubulin staining, and therefore it is mainly determining axonal length. The Calcein AM assay seems like a better assay for axon integrity. However, it looks like this assay was only used in a limited number of experiments. The authors are invited to discuss their findings in light of this concern. This is particularly important, for example, for the interpretation of the results of figure 6A when the effect of CHX is assed and Figure 8-9 when using caspase inhibitors that are protecting cell bodies and therefore axons as well.

Specific concerns about data and interpretation

1-The segment-dependence of the magnitude of response to BDNF (Fig. 2) is interesting, but one wonders if this is a BDNF-specific feature, i.e., an observation not seen with NGF? Or is the explanation simply that cervical and lumbar DRG are simply larger as limb innervation DRG contain larger number of neurons?

2- In Fig. 3A, it is unclear why there is strong Annexin V positive staining seen in the center of the explants in NGF maintained condition (Ctrl) and why this disappears after NGF deprivation.

3-In Fig. 5C, it seems like EGTA addition is affecting axons even in the presence of NGF. This complicates the overall interpretation of this experiment.

4- In Fig. 9C, the results with detection of cleaved caspase-3 are interesting and point to a potential difference between the NGF and BDNF deprivation pathways. An important consideration here is whether the time-course of degeneration after BDNF deprivation is simply slower. It would be good to examine later timepoints to confirm the results.

5- The authors should clarify if they conducted experiments with explants obtained independently from different mice (as compared to three pups from the same mom).

The study would be benefit from additional reference to work in vivo, in particular those documenting the comparatively small effect of Bdnf deprivation on the number of surviving DRG neurons, by contrast with cranial sensory ganglia. The inclusion of such references -the literature of the 90's offers a vast choice- would further help the reader, in addition to the indications mentioned in the text about the respective proportion of neurons expressing TrkB and TrkA: the

survival promoting activity of BDNF is quite modest for DRG compared with NGF, but this the opposite for some cranial sensory ganglia. In a similar vein, given the interesting results indicating the involvement of BAX in, it would be useful to mention in vivo work on cranial sensory ganglia using mice lacking both Bdnf and Bax (PMID: 15464571). The rescue effects of NAC upon BDNF deprivation are quite small and whilst the results are important and are well worth including, the Discussion should acknowledge that the effects of NAC are modest.
